# Perinatal Mortality among All Deliveries in a Tertiary Care Center: A Descriptive Cross-sectional Study

**DOI:** 10.31729/jnma.6691

**Published:** 2021-11-30

**Authors:** Saraswoti Kumari Gautam Bhattarai, Roshna Ghimire, Sapana Duwadi, Rabin Khadka, Kanchan Gautam

**Affiliations:** 1 Karnali Academy of Health Sciences, Jumla, Nepal; 2 Province Health Directorate, Karnali Province, Surkhet, Nepal; 3 Hospital for Advanced Medicine & Surgery, Mandikhatar, Kathmandu, Nepal

**Keywords:** *cause*, *incidence*, *perinatal death*, *trend*

## Abstract

**Introduction:**

Perinatal mortality comprises the number of stillbirths and death of newborns within seven days of life which is the main contributor to infant and maternal mortality. The aim of this study was to find out the prevalence of perinatal mortality among all the deliveries in a tertiary care center of a remote part of Nepal.

**Methods:**

This was a descriptive cross-sectional study conducted in a tertiary care center located in Jumla among 3798 deliveries (childbirth) from August 2014 to April 2020. Ethical approval was taken from the institutional review committee (2076/2077/05) of the same institution. A convenience sampling technique was used and the data were collected from the medical record section and then entered and analyzed in Statistical Package for Social Sciences version 16. Point estimate at 95% Confidence Interval was calculated along with frequencies and percentages for binary data.

**Results:**

The prevalence of perinatal mortality was 187 (4.92%) (4.23-4.60 at 95% Confidence Interval) among 3798 deliveries. Regarding the primary causes; the highest proportion was intrapartum hypoxia 62 (33.3%), spontaneous preterm labor 40 (21.5%), and congenital anomalies 38 (20.4%). Similarly, about the final cause; the highest proportion was birth asphyxia 64 (34.2%), intrauterine fetal death 51 (27.3%), congenital anomalies 35 (18.7%), and complication of prematurity 32 (17.1%).

**Conclusions:**

The perinatal mortality was quite high in this study with respect to similar studies done in other countries. The finding of this study showed that quality antenatal care with rural ultrasound service is essential to reduce the causes of perinatal mortality.

## INTRODUCTION

Perinatal deaths are pregnancy losses occurring after 22 weeks of gestation (stillbirths) and deaths of live births within seven days of life.^1^ Stillbirth and prematurity contributed significantly to perinatal mortality.^2^

Although neonatal mortality is declining in Nepal, it will take another 50 years for the poorest group to attain 2030 every newborn action plan target. Reducing the disparities for maternal and neonatal care will reduce mortality among the poorest families.^3^ Perinatal mortality is an important indicator of maternal care, maternal health, and nutrition, and also reflects the quality of obstetric and pediatric care available.^1^

This study aimed to find out the prevalence of perinatal mortality in a tertiary care center in a remote area of Nepal.

## METHODS

A descriptive cross-sectional study was used to find out the prevalence of perinatal death in Karnali Academy of Health Sciences teaching hospital in Jumla, which is a tertiary care facility in Karnali Province. The data were collected from 2014 August to 2020 April. This research was conducted after getting ethical approval from the institutional review committee (2076/2077/05) of the Karnali Academy of Health Sciences. The sample size was calculated using the following formula:

n = Z^2^ × p × q / e^2^

  = (1.96)^2^ × 0.5 × (1-0.5) / (0.02)^2^

  = 2401

Where,

n= minimum required sample size
Z= 1.96 at 95% Confidence Interval (CI)
p= 50%, for maximum sample size calculation
q= 1-p
e= margin of error, 5%

The calculated minimum sample size is 2401. We included 3798 deliveries admitted in the maternity ward by using the convenience sampling method.

Medical records of all women who gave childbirth between gestational ages 22 weeks and 7 days of birth and whose records were available were selected. Stillborn babies with gestational age >22weeks or weight ≥500 grams and early neonatal deaths till 7 days of life were included in the study. Document review was done by using a structured questionnaire (format) based on maternal and perinatal death surveillance and response (MPDSR) form used by the government of Nepal in every health institution.

The collected data was entered and analyzed by using Statistical Package for Social Science (SPSS) version 16. Point estimate at 95% Confidence Interval along with descriptive statistics such as frequency, percentage for binary data was calculated.

## RESULTS

Among 3798 deliveries (childbirth) the prevalence of perinatal mortality was 187 (4.92%) (4.23-4.60% at 95% Confidence Interval) from 2014 August to 2020 April. Out of 187 perinatal deaths, 111 (59.4%) were fetal death (stillbirth) and 76 (40.6%) were early neonatal deaths. Likewise, among 111 fetal deaths (still birth); 51 (45.9%) were in antepartum period (macerated stillbirth), and 60 (54.1%) were in intrapartum period (fresh stillbirth).

Regarding maternal age; more than half 114 (61%) belong to 20 to 29 years, 43 (23%) were less than 20 years, 28 (15%) were 30 to 39 years and 2 (1.1%) were above 40 years. In regard to ethnicity, the majority 118 (63.1%) belonged to Brahmin/Chhetri, 39 (20.9%) were Dalit, 17 (9.1%) were Thakuri, and 13(7%) were Janajati. Similarly, about residence; most of 156(83.4%) were from the Jumla district (Table 1).

**Table 1:** Maternal socio-demographic characteristics of perinatal death (n=187).

Socio-demographic Characteristics	Frequency n (%)
**Age**	
<20 years	43 (23)
20-29 years	114 (61)
30-39 years	30 (16)
**Ethnicity**	
Dalit	39 (20.9)
Janajati	13 (7)
Brahmin/Chhetri	118 (63)
Thakuri	17 (9.1)
**Residence**	
Jumla	156 (83.4)
Dolpa	3(1.6)
Mugu	12 (6.4)
Kalikot	16 (8.6)

Regarding antenatal care received by the mother; 80 (42.8%) were received and 79 (42.2%) were not received antenatal care. About the gravida; 75 (40.1%) were primigravida 62 (19.8%) were multi gravida and 50 (13.4%) were grand multigravida. Likewise, about parity; 85 (45.5%) were nulliparous, 87 (46.5%) were multiparous and 15 (8.0%) were grand multiparous. About the mode of delivery; 148 (79.1%) had a spontaneous vaginal delivery, 6 (3.2%) had a forceps delivery, 3 (1.6%) had vacuum delivery, 27 (14.4%) had a cesarean section, and 3 (1.6%) had a destructive operation (Table 2).

**Table 2:** Obstetrical Characteristics of Perinatal Death (n=187).

Obstetrical Characteristics	n (%)
**Antenatal care**	
Yes	80 (42.8)
No	79 (42.2)
Don't know	28 (15)
**Gravida**	
Primigravida	75 (40.1)
Multigravida	62 (33.2)
Grand multigravida	50 (26.7)
**Parity**	
Primipara	39 (38.2)
Multipara	48 (47.1)
Grand multipara	15 (14.7)
**Mode of Delivery**	
Spontaneous vaginal delivery	148 (79.1)
Forceps	6 (3.2)
Vacuum	3(1.6)
Cesarean Section	27 (14.4)
Destructive operation	3(1.6)

In regard to birth weight; 27 (14.4%) were less than 1000 g, 31 (16.6%) were 1000-1500 gm, 48 (25.7%) were 1500-2499 gm, 80 (42.8%) were 2500-3999 gm, and 1 (.5%) were more than or above were 4000 gm. Regarding the gestation; 95 (50.8%) were preterm, 63 (33.7%), 63 (33.7%) were term, 15 (8.0%) were postterm, and 14 (7.5%) were query. About the sex; 102 (54.5%) were male, 85 (45.5%) were female and 1 (0.5%) were ambiguous. Among perinatal death; 182 (97.3%) were singleton and 5 (2.7%) were multiple (Table 3).

**Table 3:** Neonatal parameters of perinatal death (n=187).

Neonatal parameters	n (%)
**Birth weight (grams)**	
<1000	31 (16.6)
1000-1500	48 (25.7)
1500-2499	80 (42.8)
2500-3999	1 (.5)
>=4000	27 (14.4)
**Gestational Age**	
Preterm	95 (50.8)
Term	63 (33.7)
Post-term	15 (8.0)
Query	14 (7.5)
**Sex**	
Male	102 (54.5)
Female	84 (44.9)
Ambiguous	1 (0.5)
**Delivery**	
Singleton	182 (97.3)
Multiple	5 (2.7)

Regarding the primary causes; 40 (21.5%) had spontaneous preterm labor, 62 (33.3%) had intrapartum hypoxia, 15 (8.1%) had antepartum hemorrhage (APH), 5 (2.7%) had the hypertensive disorder, 11 (5.9%) had an infection, 38 (20.4%) had congenital anomalies, 2 (1.1%) had intrauterine growth restriction (IUGR), 1 (0.5%) had trauma, 22 (11.8%) had an unexplained intrauterine cause, 18 (9.7%) had intrauterine fetal death (IUFD), and 4 (2.2%) had other causes. Among 4 other causes; one had CPD with prolonged labor, one fall injury, one hand prolapse, and one had oligohydramnios. Concerning the final cause of perinatal death; 64 (34.2%) had birth asphyxia, 10 (5.3%) had septicemia, 5 (2.7%) had pneumonia, 32 (17.1%) had complications of prematurity, 35 (18.7%) had congenital anomalies, 2 (1.1%) had hypothermia, 51 (27.3%) had IUFD, and 4 (2.1%) had other causes. About the Wigglesworth Classification of perinatal death; 52(27.8%) were normally formed macerated stillbirth, 30 (16.0%) were lethal congenital malformation, 36 (19.3%) were condition associated with prematurity, 61 (32.6%) were asphyxia condition includes fresh stillbirth, and 8 (4.3%) were others. Among 8 other causes; 3 had Pneumonia, 2 had septicemia, 2 had status epilepticus, and one had hypoglycemia. (Table 4).

**Table 4:** Causes of perinatal death (n=187).

Primary Cause death	n (%)
Spontaneous preterm labor	40 (21.4)
Intrapartum hypoxia	57 (30.5)
APH	15 (8.0)
Hypertensive disorder	5 (2.7)
Infection	11 (5.9)
Congenital anomalies	36 (19.3)
IUGR	2 (1.1)
Trauma	1 (0.5)
IUFD	18 (9.6)
Unexplained intrauterine cause	22 (11.8)
Others	12 (6.4)
**Final cause of death**	
Birth asphyxia	62 (33.2)
Septicemia	9 (4.8)
Pneumonia	5 (2.7)
Complication of prematurity	30 (16)
Congenital anomalies	35 (18.7)
Hypothermia	2 (1.1)
IUFD	47 (25.1)
Other	15 (8)
**Wigglesworth Classification of death**	
Normally formed macerated stillbirth	52 (27.8)
Lethal congenital malformation	30 (16)
Condition associated with prematurity	36 (19.3)
Asphyxia condition includes fresh stillbirth	61 (32.6)
Other	8 (4.3)

**Figure 1:**
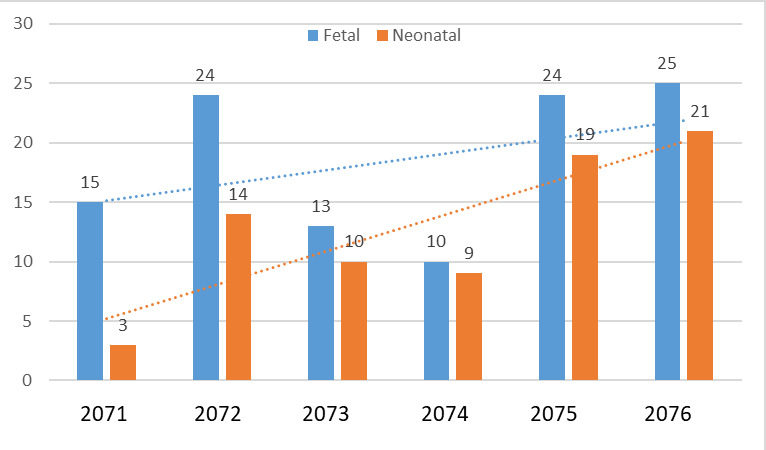
The trend of Perinatal death (N=187).

Perinatal death in 2071 (2014 to 2015) was 18 (9.6%), in 2072 (2015-2016) was 38 (20.3%), in 2073 (2016-2017) was 23 (12.3%), in 2074 (2017-2018) was 19 (24.6 %), in 2075 (2018-2019) was 43 (10.2%), and in 2076 (20192020) was 46 (23.0%) (Figure 1).

## DISCUSSION

Among 3798 childbirth in Karnali Academy of Health Sciences, the prevalence of perinatal mortality was 187 (4.92%) from 2014 August to 2020 April. A study done in Arghakhanchi district hospital showed the perinatal mortality rate was 32.2 per 1,000 births,^4^ which is nearly similar to our study. According to a cross-sectional descriptive study done at the maternity hospital in Duhok, the perinatal deaths were 496 (1.22%),^5^ which is less than our study. Likewise, the perinatal mortality rate was 10.83 per 1000 total births in Tanzania,^6^ which is also less than our finding. Among 187 perinatal deaths, 111 (59.4%) were stillbirth and 76 (40.6%) were early neonatal deaths. A study done in Duhok showed 268 (54%) early neonatal deaths and 228 (45.5%) stillbirth,^5^ which is in contrast to this study finding. Similarly, the perinatal mortality rate was 13.6 per 1000 total births in Georgia. Among them, stillborn were 9.1 per 1000 total births and early neonatal deaths were 4.5 per 1000 live births,^7^ which is lower than our study findings. In this study among 111 fetal deaths (stillbirth), 51(45.9%) were in the antepartum period (macerated stillbirth), and 60 (54.1%) were in the intrapartum period (fresh stillbirth). In Georgia, the majority of stillbirths (85%) died in antepartum and 9% were in the intrapartum period.^7^ A study done in Duhok showed that the early neonatal deaths were 268 (54%) and stillbirth was 45.5%. Among stillbirths, 151 (30.5%) were fresh, and 77 (15.5%) were macerated stillbirths,^8^ that is nearly similar to the finding of this study.

Regarding the maternal age; more than half 114(61%) belong to 20 to 29 years, 43(23%) were less than 20 years, 28(15%) were of 30 to 39 years and 2(1.1%) were above 40 years. In Duhok, 52.6% were 20-29 and 35.3% were 30-39 years old.^8^ Another study done in Sikkim revealed that 57.5% were of 18-25 years and 32.5% were above 35 years of age had more perinatal deaths,^9^ which was different from our findings. This difference may be due to the difference in sociocultural background. Similarly, about the residence of mother, most of 156 (83.4%) were from Jumla, and least 3 (1.6%) were from Dolpa district.

Regarding the obstetrical characteristics and perinatal death; 80 (42.8%) received, 79(42.2%) did not receive antenatal care. According to Jhansi, India; 154 (83.70%) had not done an antenatal check-up and 30 (16.30%) had done antenatal check-ups,^10^ which is in contrast to this study. About the gravida of mother; 75 (40.1%) were primigravida, 62 (19.8%) were multi gravida and 50 (13.4%) were grand multigravida. A study done in Jhansi showed that the perinatal mortality in multigravida is relatively higher than primigravida,^10^ which looks similar to this study. Likewise, about parity of mother; 85(45.5%) were nulliparous, 87(46.5%) were multipara and 15(8.0%) were grand multiparous. According to the study of Sikkim, more perinatal losses occurred in multiparous women (61%) as compared to primiparous women (39%),^9^ that is in contrast to this study found. In this study the majority (79.1%) had a normal vaginal delivery and 14.4% had a cesarean section. According to a study conducted in Iran, 73.1% had a normal vaginal delivery and 26.9% had a cesarean section.^11^ This shows that a similar finding was found in vaginal delivery and contrast in cesarean section.

In regard to birth weight 27 (14.4%) were less than 1000 g, 31 (16.6%) were 1000-1500 gram, 48(25.7%) were 1500-2499 gm, 80 (42.8%) were 2500-3999 gm, and 1 (.5%) were more than or above were 4000 gm. One study conducted in Nigeria depicted the low birth weight baby 55 (38.46%), normal birth weight 73 (51.04%), Macrosomia 13 (9.09%).^12^ Regarding the gestation 95 (50.8%) were preterm, 63 (33.7%) were term, and 15 (8.0%) were post-term. The contrast finding was found in a study conducted in one Medical College and Teaching Hospital, Kathmandu that is 81.25% were preterm births while 18.75% were term.^2^ Another study conducted by Rai et al showed perinatal deaths more at gestational age <34 weeks and at >40 weeks,^9^ which has different findings with this study. About the sex of the baby; 102 (54.5%) were male and 85 (45.5%) were female. A study conducted in Kathmandu showed 50% were male and 50% female.^2^ In this study 182 (97.3) were singleton and 5 (2.7%) were multiple births which is a contrast result with the study finding of Bangladesh that is perinatal mortality was approximately twice among twin babies in comparison with single births.^13^

Regarding the primary causes; 40 (21.5%) had spontaneous preterm labor, 62 (33.3%) had intrapartum hypoxia, 15 (8.1%) had antepartum hemorrhage (APH), 5 (2.7%) had the hypertensive disorder, 11 (5.9%) had an infection, 38 (20.4%) had congenital anomalies, 2 (1.1%) had intrauterine growth restriction (IUGR), 1 (.5%) had trauma, 22 (11.8%) had an unexplained intrauterine cause, 18 (9.7%) had intrauterine fetal death (IUFD), and 4 (2.2%) had other causes. Among 4 other causes; one had CPD with prolonged labor, one fall injury, one hand prolapse, and one had oligohydramnios. In a study done in South Africa most common primary causes were hypertension and obstetric hemorrhage. Among them; spontaneous preterm labour 39.6%, infections 2.1%, antepartum haemorrhage 23.7%, intrauterine growth restriction 1%, hypertensive disorders 31.7%, fetal abnormality 7.3%, trauma 0.7%, intrapartum asphyxia 55%, maternal disease 1.8%, miscellaneous 0.7%, unexplained intrauterine death 34.6%,^14^ which result looks similar to this study finding. Another study conducted in India revealed that prematurity was the major cause of perinatal mortality. Obstetrical complications associated with perinatal mortality is antepartum hemorrhage (4.35%), eclampsia (10.32%), obstructed labour (8.15%), mal-presentation (11.96%).^7^ A study done in Georgia, depicts that the cause of death of the majority (80%) of stillbirths was unknown.

The most common causes of death for stillbirths were maternal conditions (7.8%) and complications of the placenta and the umbilical cord (5.2%). Congenital malformations had in 2.6% of stillbirths.^7^ In regard to the final cause of perinatal death; 64 (34.2%) had birth asphyxia, 10 (5.3%) had septicemia, 5 (2.7%) had pneumonia, 32 (17.1%) had complications of prematurity, 35 (18.7%) had congenital anomalies, 2 (1.1%) had hypothermia, 51 (27.3%) had IUFD, and 4 (2.1%) had other causes. A study conducted in Georgia cause of death was preterm delivery (58%), followed by congenital malformations (23%), birth asphyxia (7%), and infections (7%).^7^

Regarding the Wigglesworth Classification of death; 52 (27.8%) were normally formed macerated stillbirth, 30 (16.0%) were lethal congenital malformation, 36 (19.3%) were condition associated with prematurity, 61 (32.6%) were asphyxia condition includes fresh stillbirth, and 8 (4.3%) were others.

In regard to the trend of perinatal death; 18 (9.6%) was in 2014 to 2015 was 18 (9.6%), in 2015-2016 was 38 (20.3%), in 2016-2017 was 23 (12.3%), in 2017-2018 was 19 (24.6%), in 2018-2019 was 43 (10.2%), and in 2019-2020 was 46 (23%). Perinatal death surveillance of Sri Lanka showed that the perinatal death rate was 9.94 per 1000 total births in 2014, 7.98 in 2015; 8.08 in 2016; and 6.79 in 2017. From 2014 to 2017, there was a declining trend of perinatal mortality.^15^ A study of Ethiopia showed a slight reduction trend with some ups and downs in-between years of publication.^16^

## CONCLUSIONS

The perinatal mortality was quite high in this study with respect similar studies done in other countries. The finding of this study showed that quality antenatal care with rural ultrasound service is essential to reduce the causes of perinatal mortality. Regarding the primary causes of perinatal death; the highest proportion had intrapartum hypoxia, spontaneous preterm labour, and congenital anomalies. Similarly, about the final cause of perinatal death, the highest proportion had birth asphyxia, IUFD, congenital anomalies, and complications of prematurity. Likewise, about the Wigglesworth classification, the highest proportion had asphyxia conditions including stillbirth, followed by normally formed macerated stillbirth, conditions associated with prematurity, and lethal congenital malformation. In conclusion, quality antenatal care with rural ultrasound service is essential to identify and reduce the different causes of perinatal death. Therefore, quality maternity care service is required from gross root level to improve the utilization of quality maternity service and reduce the perinatal mortality in Karnali Province.
